# Real-World Long-Term Engagement With a Mobile App Intervention to Improve Self-Management of Type 2 Diabetes Mellitus in China (SMARTDiabetes): Mixed Methods Study

**DOI:** 10.2196/76699

**Published:** 2026-03-03

**Authors:** Xuanchen Tao, David Peiris, Yuxia Ma, Yaoshen Zhang, Hongyi Song, Limin Mao, Puhong Zhang

**Affiliations:** 1 The George Institute for Global Health, University of New South Wales, Sydney, Australia; 2 Children’s Hospital at Westmead Clinical School, Faculty of Medicine and Health, The University of Sydney, Sydney, Australia; 3 School of Public Health, Hebei Medical University, Shijiazhuang City, Hebei Province, China; 4 Hebei Key Laboratory of Environment and Human Health, Shijiazhuang City, Hebei Province, China; 5 Luquan Center for Disease Control and Prevention, Shijiazhuang City, Hebei Province, China; 6 The George Institute for Global Health, Beijing, China; 7 Center for Social Research in Health, University of New South Wales, Sydney, Australia; 8 Beijing Physical Examination Center, No 59 Beiweilu, Xicheng District, Beijing, 100050, China, 86 13691270366

**Keywords:** digital behavior change intervention, mHealth, engagement, type 2 diabetes mellitus, China, primary health care, implementation

## Abstract

**Background:**

Despite the high prevalence of type 2 diabetes mellitus in China, few patients are achieving adequate management targets. While digital behavior change interventions (DBCIs) are promising solutions, sustained long-term user engagement with these new technologies remains challenging.

**Objective:**

The objectives of this study were to (1) describe the long-term user engagement with the SMARTDiabetes app, (2) explore the associations between user engagement and trial effectiveness, and (3) identify main influencing factors of user engagement.

**Methods:**

A mixed methods process evaluation was conducted alongside the 2-year SMARTDiabetes trial to assess long-term user engagement with the DBCI, investigate its association with trial effectiveness, and explore its influencing factors. Quantitative data included clinical outcomes and app usage data; qualitative data included an expert review of the DBCI and semistructured interviews with key stakeholders. Findings were synthesized using Michie’s conceptual framework for engagement with DBCIs.

**Results:**

A total of 1038 participants were included in the quantitative analyses, and 12 in-depth interviews were conducted in June 2020. The mean proportion of monthly active users was 46.4% (SD 7.8%), with a gradual decline from 56.3% at the start (April 2018) to 42.2% at the end of trial (July 2019). The proportion of sustained active users was 32.9% (342/1038), with rural users showing a significantly higher rate than urban users (38.3% vs 27.3%; *P*<.001). We found a strong link between user engagement and the effectiveness in achieving primary health outcome (risk ratio 1.37, 95% CI 1.21‐1,55; *P*<.001) and a clear dose-response relationship between the level of app interactions and degree of improvement in the health outcome. Key drivers of user engagement included 6 behavior change techniques (ie, reward, feedback, reminders, goal setting, self-monitoring, and social support features), 4 implementation facilitators (credibility features, professional support, guidance, and ease of use), and 5 contextual contributors (younger age, better education, greater smartphone literacy, closer patient-doctor relationship, and stronger primary health care facilities commitment).

**Conclusions:**

Long-term, sustained engagement with a mobile app intervention for type 2 diabetes mellitus self-management is achievable in the primary health care setting in China. Higher engagement is associated with better health outcomes. The influencing factors identified in the SMARTDiabetes study provide valuable insights for developing targeted strategies to enhance user engagement with DBCIs and improve overall program effectiveness.

## Introduction

China has the largest number of patients with type 2 diabetes mellitus (T2DM) globally. The Chinese government has launched a series of health reforms since 2009, aiming to improve the management of diabetes by strengthening primary health care (PHC) [1]. In China, PHC facilities are typically the first point of contact for patients with the national health system, and individuals with T2DM receive most of their health care services at these facilities. PHC facilities are organized into 2 tiers, with grassroots-level facilities managed under the supervision of center-level facilities. In urban areas, community health stations are managed by community health centers, whereas in rural areas, village clinics are managed by township health centers [1]. Despite the reforms, the rates of awareness (36.7%), treatment (32.9%), and control (50.1%) of diabetes remained low [2]. The study projected that the suboptimal management of diabetes in China will lead to a rapid increase in economic burden, with the total costs rising from US $250.2 billion in 2020 to US $460.4 billion by 2030 [3]. These findings underscore the urgent need for better and innovative T2DM management strategies.

To address this challenge, we developed a multifaceted digital behavior change intervention (DBCI) and evaluated it in a 24-month cluster randomized controlled trial in 80 PHC facilities (40 rural villages and 40 urban communities) in Shijiazhuang City, Hebei Province, China [4,5]. The SMARTDiabetes intervention was underpinned by a mobile health (mHealth) system comprising three integrated components: (1) an app for patients and lay family members, also known as family health promoters (FHPs), who were invited to assist patients in managing their health conditions; (2) an app for PHC providers, which provided e-clinical decision support and access to a daily e-log containing data entered by patients; and (3) a web-based dashboard for local health administrators or governors, which generated real-time data-monitoring reports of key performance indicators (Figure S1 in Multimedia Appendix 1). The primary clinical outcome was the difference in proportion of patients achieving at least 2 American Diabetes Association “ABC” management targets ([A] glycated hemoglobin [HbA_1c_] <7.0%, [B] blood pressure (BP) <140/80 mm Hg, and [C] low-density lipoprotein cholesterol <2.6 mmol/L) at 24 months. Overall, the SMARTDiabetes intervention significantly improved the primary outcome (intervention group: 35.9% vs usual care: 29.9%; *P*=.03). However, we observed significant heterogeneity in the primary outcome between rural and urban settings; the same intervention was significantly more effective in rural areas than in urban areas (rural—intervention group: 42.6% vs usual care: 31.0%; urban—intervention group: 27.9% vs usual care: 28.6% [*P*=.02 for heterogeneity]) [5].

Engagement with DBCIs, defined by (1) the extent (eg, amount, frequency, duration, and depth) of usage and (2) a subjective experience characterized by attention, interest, and affect [6], is logically a precondition for effectiveness [7-11]. Many studies on diabetes self-management apps have shown a positive association between user engagement and intervention effectiveness [12-15]. However, in practice, low clinical uptake, poor user engagement, and sustaining long-term participation are common challenges for these DBCIs [16-18]. The SMARTDiabetes trial is the longest (2 years) mobile app intervention for improving T2DM self-management with a rigorous research design (clustered randomized controlled trial) and a decent sample size (about 2000 patients) in the real-world PHC setting. Therefore, evaluating the long-term user engagement and understanding its influencing factors are crucial for optimizing the DBCIs design and implementation. These insights can help develop targeted strategies to enhance user engagement and improve overall program effectiveness [11,19].

In this study, we adopted a mixed methods multidimensional approach to assess both the subjective and objective measures of user engagement [6,11]. The aims of this study were to (1) describe the long-term user engagement with the SMARTDiabetes app, (2) explore the associations between user engagement and trial effectiveness (ie, the “ABC” biomarkers predetermined as the primary and secondary trial outcomes), and (3) identify main influencing factors of user engagement.

## Methods

### Study Design

In this mixed methods process evaluation, we conducted a descriptive analysis of user engagement with the SMARTDiabetes app over 2 years and a post-hoc analysis of its association with the trial effectiveness. In addition, to identify main influencing factors with the user engagement and trial effectiveness, we conducted a rapid theory-informed expert review of the DBCI tools, followed by 12 semistructured interviews to key stakeholders (ie, selected PHC providers, patients, and their FHPs in the intervention arm) conducted in June 2020, and concluded with a synthesis of evidence to explain the significant geographic heterogeneity in trial effectiveness observed in our previous publication (see Figure S2 in Multimedia Appendix 1 for timeline for data collection) [5]. The mixed methods study conformed with the GRAMMS (Good Reporting of A Mixed Methods Study) checklist [20].

### Setting and Participants

The study recruitment was conducted at 80 PHC facilities (40 rural and 40 urban) in Shijiazhuang city, China, from August 2017 to December 2017. A full description of selection criteria for PHC facilities has previously been published [5]. PHC providers invited participants from the diabetes management list within the routine registration system for screening. This list included all identified patients with diabetes within the service range of the PHC facilities. The recruitment inclusion criteria were adults who (1) were diagnosed with T2DM, (2) were aged 40 years or older, (3) had a HbA_1c_ level of 7.0% or higher, (4) had access to the internet via a smartphone (confirmed verbally), either independently or through a designated family member, and (5) provided informed consent. Patients were excluded if they (1) had severe physical or psychological injury or illness, (2) were unable to attend the site visit or consciously answer questions, (3) were women in the process of or planning for pregnancy or breastfeeding, or (4) participated in any other clinical trial within the previous 6 months.

### SMARTDiabetes Intervention

**Figure 1.**
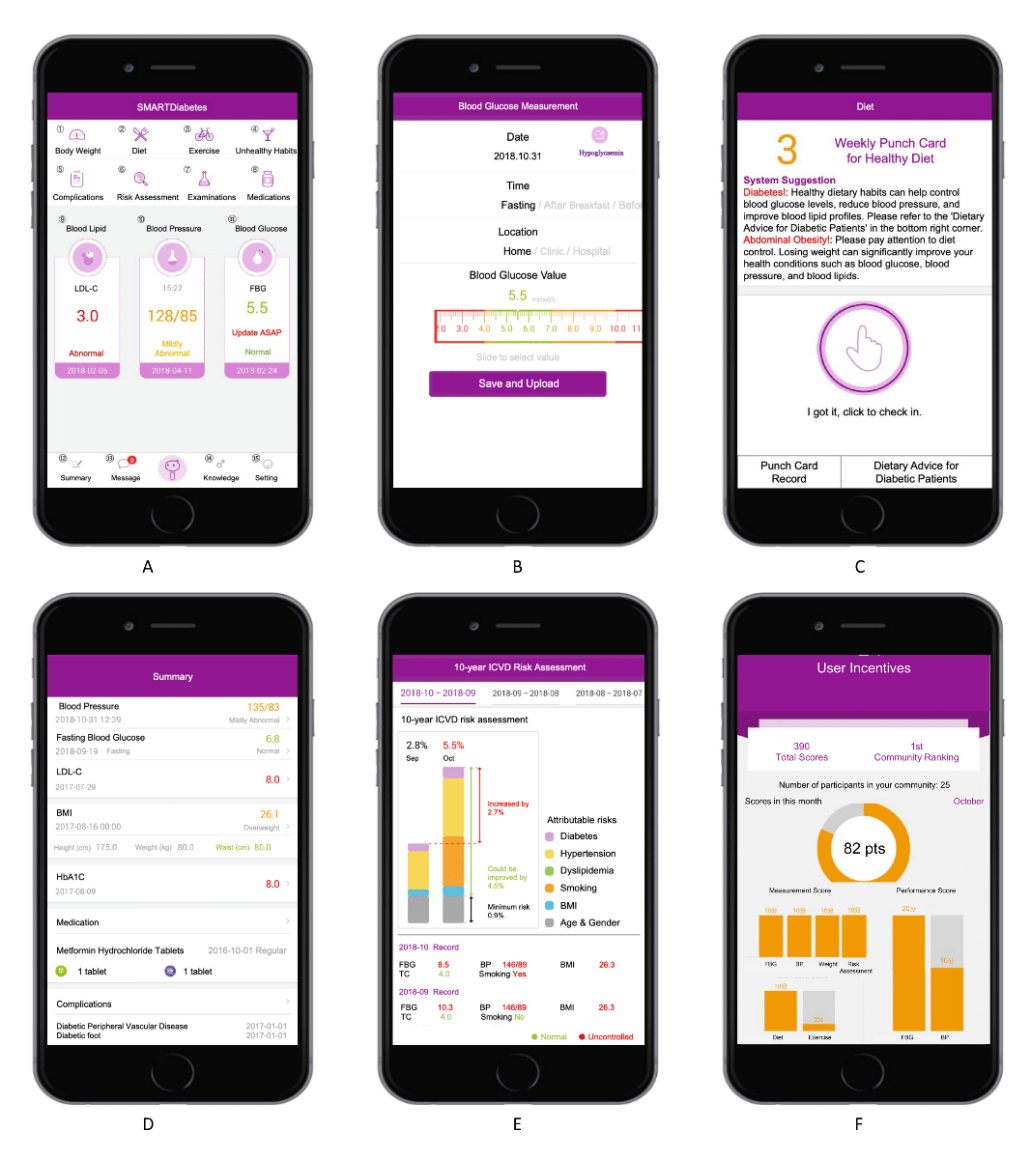
Main functions of the SMARTDiabetes app.

Based on a previous mixed methods study that systematically explored major barriers to and facilitators of PHC management of T2DM in China [21], our team developed the SMARTDiabetes app adopting a user-centered design approach [4,5]. In brief, the SMARTDiabetes app incorporates recommendations from Chinese national and international guidelines to support disease self-monitoring, adoption of healthier lifestyles, better medication adherence, and prevention of diabetes complications [4]. As illustrated in Figure 1, a total of 15 functions are displayed on the main landing page (Figure 1A). Four functions, “Blood glucose,” “Blood pressure,” “Blood lipid,” and “Body weight” (Figure 1B), are for disease self-monitoring, where users can upload their latest data to receive immediate in-app feedback with result interpretation, improvement suggestions (goal setting), and relevant healthy literacy materials. The next 2 functions, “Diet” and “Exercise” (Figure 1C), are assisted by a digital punch card (*Da Ka*—clocking registration) system, which sends reminders for consuming healthier diets and engaging in appropriate physical activities. A further 5 functions, “Unhealthy lifestyles,” “Health status summaries,” “Physical examinations and lab tests,” “Medications,” and “Diabetes complications” (Figure 1D), serve as a medical record, enabling the tracking of unhealthy practices, medication adherence, and disease progression. The 12th function—“iCVD risk assessments” (Figure 1E) shows onset probabilities of ischemic cardiovascular diseases (iCVDs) including stroke within the following 10-year period, accompanied by a forecasted, monthly adjusted risk progression curve, on the basis of users’ latest self-monitored data entry. This risk forecast model was constructed by the research team, using the risk assessment tools of Wu and colleagues [22]. The final 3 functions include 2 administrative functions (“Settings” and “Messages”) and 1 preloaded health literacy function (“Knowledge and skills bank”).

The reward program (Figure 1F) allows users to earn and accumulate reward points by interacting with the 6 core app functions (ie, “Blood glucose self-monitoring,” “Blood pressure self-monitoring,” “Body weight,” “Diet punch card,” “Exercise punch card,” and “iCVD risk assessment quiz”). Users can earn up to 100 points per month (see detailed scoreboard in Table S1 in Multimedia Appendix 1) and redeem their points for small gifts of little monetary value, such as soap, towels, or laundry powder, from their respective PHC providers in the communities.

Before the trial commenced, the research team conducted a comprehensive training session for the 40 PHC providers in the intervention arm, covering downloading, installing, and using both the patient and provider versions of the SMARTDiabetes app. Subsequently, PHC providers organized training sessions for their enrolled patients and the patients’ FHPs. To facilitate and standardize these sessions, a PowerPoint slide deck and an instruction brochure were developed (Figure S2 in Multimedia Appendix 1).

### User Engagement Metrics

Deidentified app usage data collected between April 2018 and July 2019, including log-in frequencies and app functions click-through counts, were extracted by the service provider (Beihang University) and sent to the research team for secondary data analysis. In addition, a series of autogenerated key performance indicators (eg, BP control rates and fasting blood glucose [FBG] self-monitoring rates), in the form of cluster-level aggregated monthly reports, were presented on the SMARTDiabetes website. The research team documented these key performance indicators at the end of each month.

User engagement was evaluated using the following four metrics. (1) The proportion of monthly active users (MAUs), defined as participants who logged into the app at least once in a certain month, which was calculated by dividing the number of MAUs by the total number of participants in the intervention arm. Sustained active users were defined as participants who maintained MAU status for at least 12 months within the 16-month assessment period (April 2018 to July 2019), while others were categorized as “inactive users.” (2) The proportion of users with FHP involvement, which was calculated by dividing the number of patients who reported receiving FHPs’ assistance in app use by the total number of participants in the intervention arm. (3) The total click-through counts per month, which was calculated by adding the number of clicks by each function. (4) The proportion of users who adhered to the recommended FBG self-monitoring frequencies per the SMARTDiabetes app’s algorithm (see the algorithm in Table S2 in Multimedia Appendix 1).

The first 2 metrics were derived from app usage data, the third metric was obtained from the final trial assessment questionnaire, and the fourth metric was a cluster-level performance indicator retrieved from the SMARTDiabetes website. Descriptive analysis was conducted using SAS software (version 9.4 or above; SAS Institute). Means and SDs were reported for continuous variables with normal distribution; medians and IQRs were reported when the data were skewed. Proportions were reported for categorical variables.

### Association Between User Engagement Levels and Trial Effectiveness

In a post hoc analysis, associations with the primary outcome at 24 months were analyzed for the following subgroups: (1) sustained active users versus inactive users, (2) users with versus without FHP involvement in app use assistance, and (3) click-through registration levels, with users grouped into quartiles based on the total number of clicks on app functions during the same period.

An inverse propensity score weighted analysis was conducted to compare the trial effectiveness across subgroups with different engagement levels. Propensity scores were developed to control for the differences in baseline demographics, medical history, and laboratory characteristics across subgroups. Propensity scores were generated by a logit regression model with covariates of age, sex, locality, HBA_1c_, FBG, systolic BP, diastolic BP, low-density lipoprotein cholesterol, education levels, duration of diabetes category, and diabetic complications. A log-binomial regression model with generalized estimation equation and adjustment for community clustering and the weighted inverse propensity score was then developed. The associations were presented as the relative risk (RR) with its 95% CIs. All analyses were performed with SAS software (version 9.4 or above; SAS Institute).

**Figure 2.**
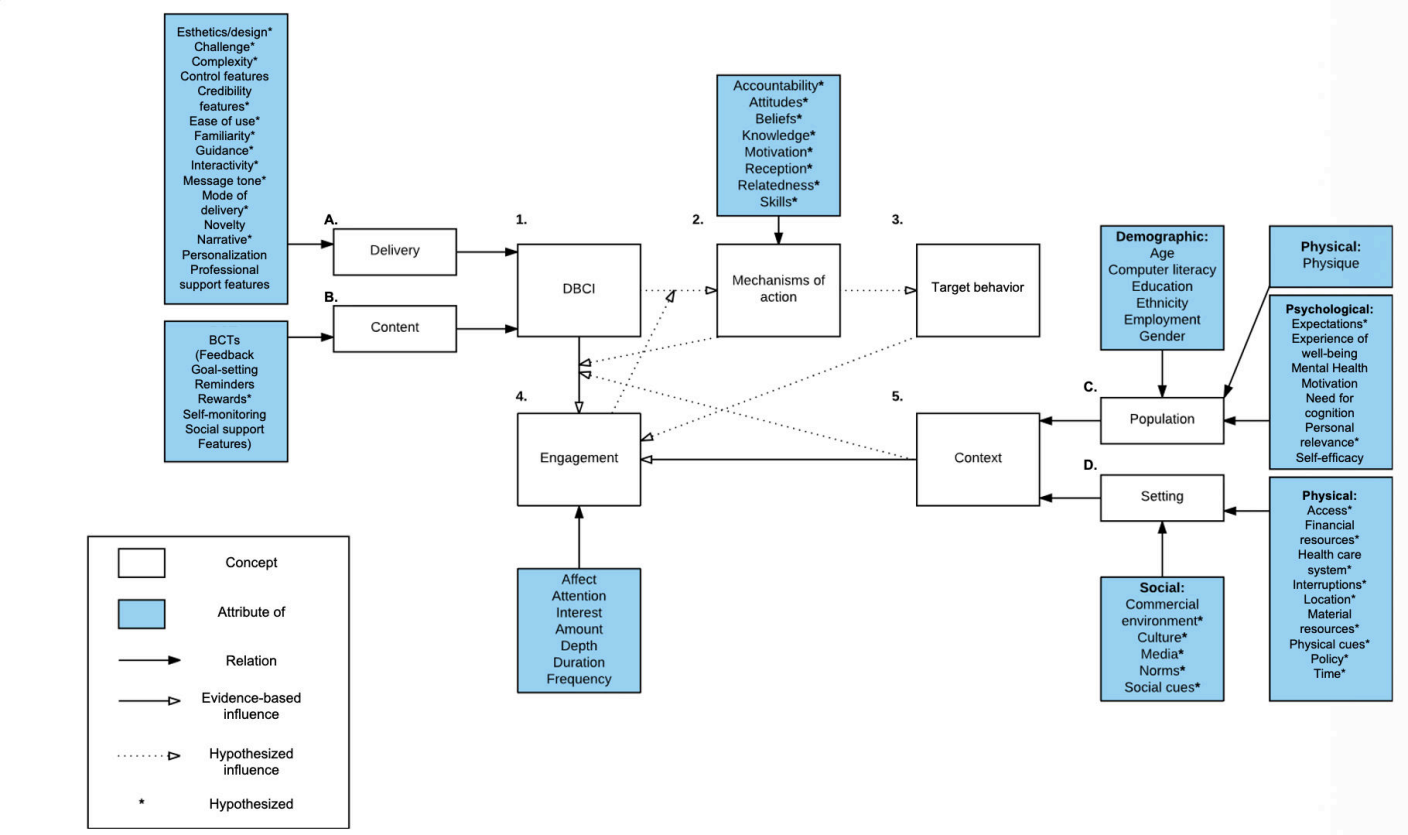
Conceptual framework of direct and indirect influences on engagement with DBCIs. BCTs: behavior change techniques; DBCI: digital behavior change intervention.

**Figure 3.**
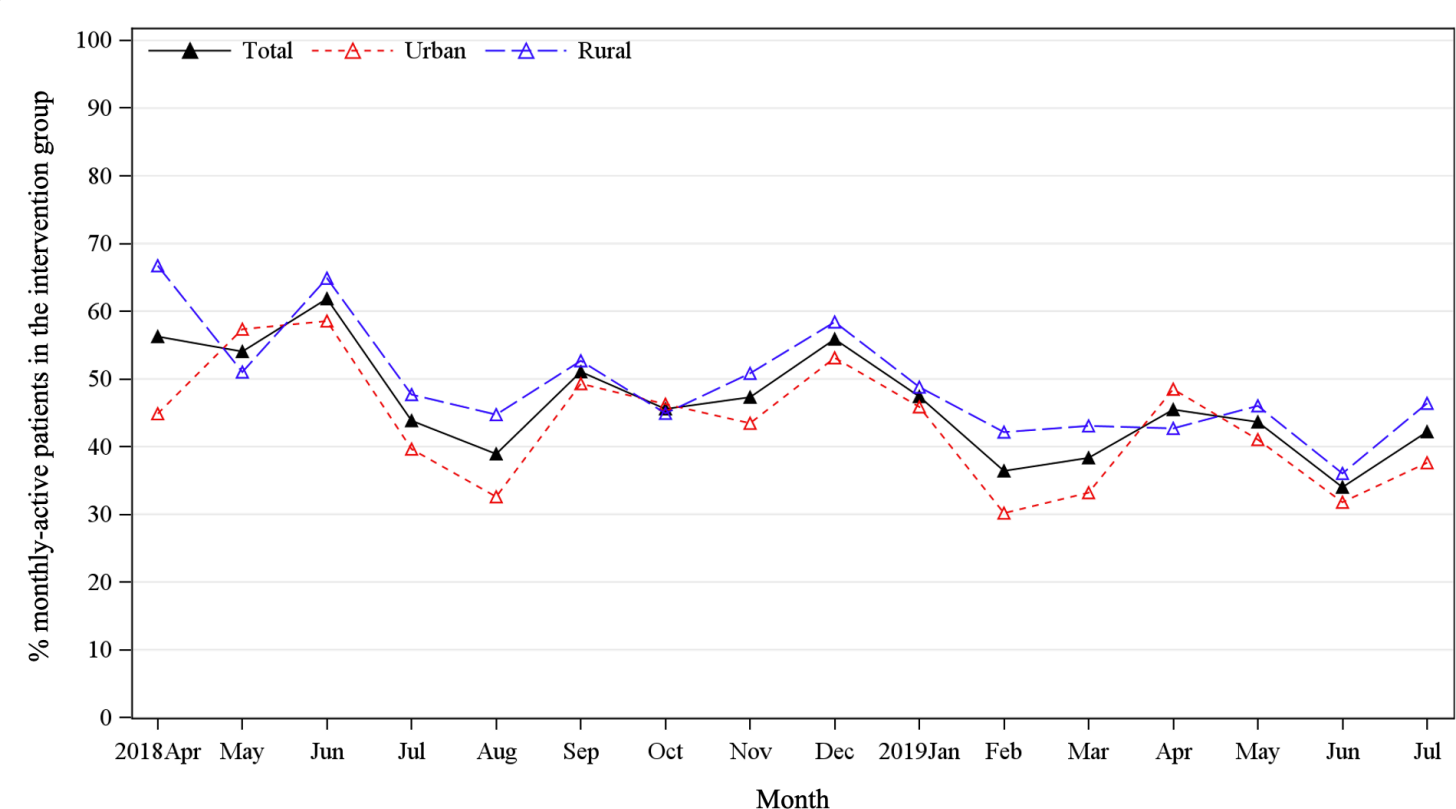
The proportion of monthly active users.

**Figure 4.**
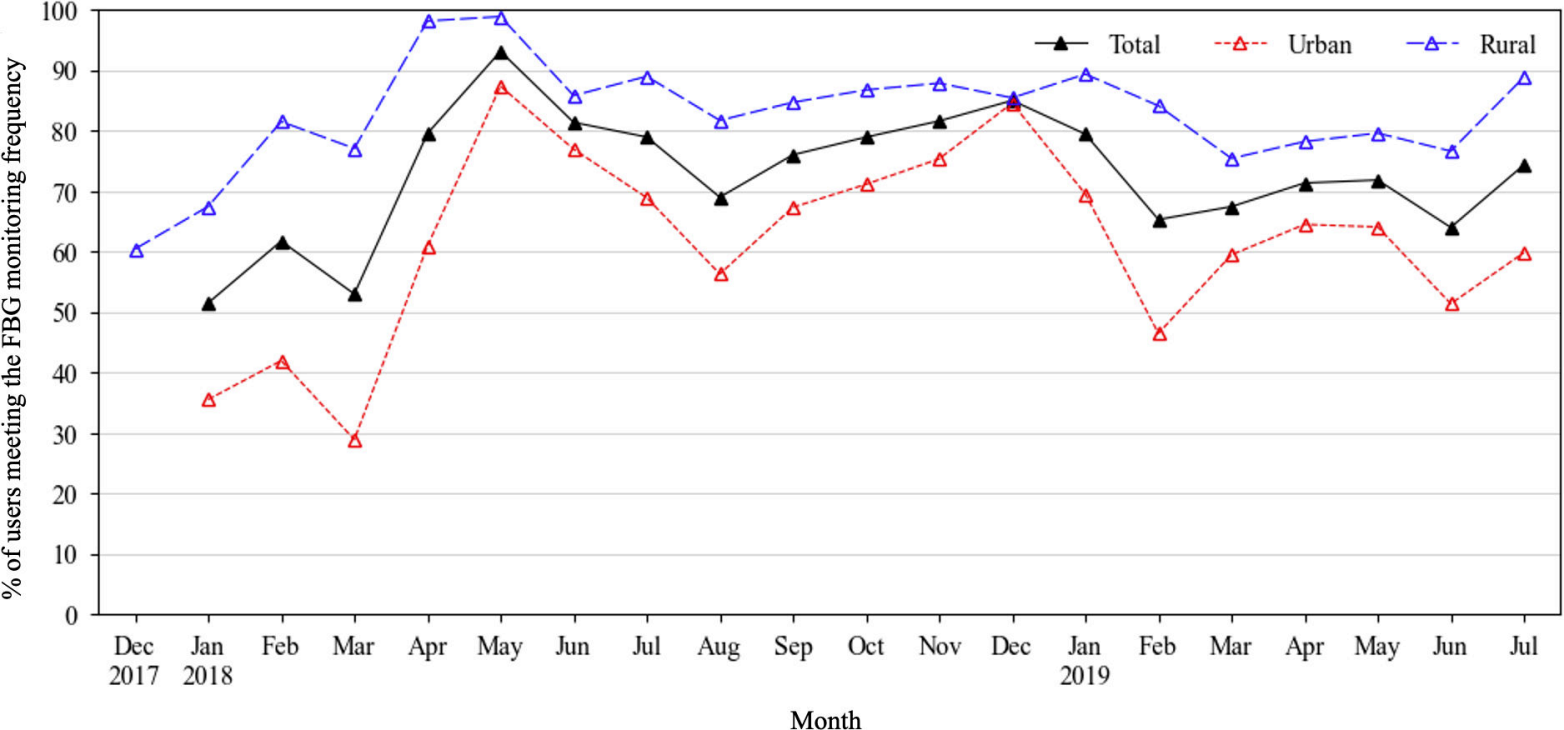
The proportion of users meeting the FBG monitoring frequency. FBG: fasting blood glucose.

To better understand the geographic heterogeneity in trial effectiveness observed in our primary results study [5], we conducted additional subgroup analyses by locality to understand their differences in PHC facilities characteristics (Figure 2D), population baseline characteristics (Figure 2C), and engagement patterns (Figures 2-4). Two-tailed *t* tests were conducted for continuous variables with normal distribution, Wilcoxon signed-rank tests were conducted for continuous variables without a normal distribution, and chi-square tests were conducted for categorical variables.

### Influencing Factors of User Engagement

After the trial, 2 qualitative activities were undertaken to explore the influencing factors for user engagement and trial effectiveness.

**Figure 5.**
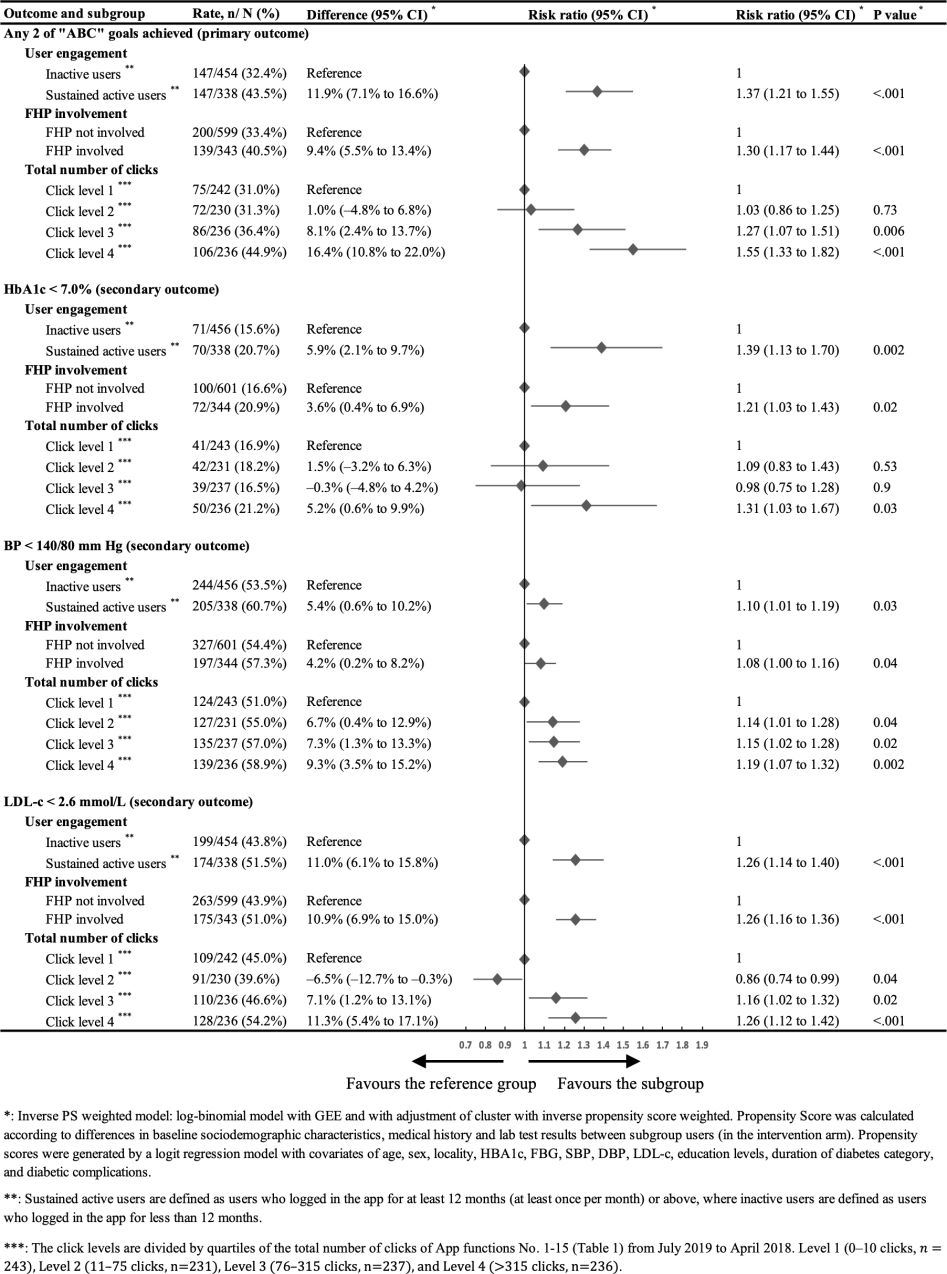
Forest plot of the effects of user engagement on main clinical outcomes. BP: blood pressure; FHP: family health promoter; HbA_1c_: glycated hemoglobin; LDL-c: low-density lipoprotein cholesterol.

1. An expert review of the DBCI including app features (DBCI content, Figure 2B) and trial implementation activities (DBCI delivery, Figure 2A) based on the conceptual DBCI engagement framework by Michie and colleagues [6]. This framework conceptualizes the potential direct and indirect influences on engagement, as well as the relationships between engagement and the effectiveness of the interventions. The behavior change techniques (BCTs) adopted by our DBCI were identified against the internationally recognized Behavior Change Technique Taxonomy (V1) [23]. Two researchers (XT and PZ) independently conducted the review. With the majority in agreement, a small amount of disagreement was resolved by discussion and a consensus was reached.
2. In-depth interviews. Interviews with key stakeholders, including selected PHC providers, patients, and FHPs in both urban and rural settings, were conducted in June 2020 to deepen our understandings of factors contributing to user engagement with the app. Semistructured, user-type specific interview guides were developed by our multidisciplinary research team, piloted with a few participants, and then revised (see posttrial interview guides in Multimedia Appendix 1). Face-to-face, one-on-one in-depth interviews, each lasting approximately 1 hour, were conducted in Mandarin Chinese by 2 team members (XT and PZ) in designated private areas. The interviews were audio-recorded and transcribed verbatim. Iterative thematic analysis was conducted by generating codes, forming themes, and further categorizing into 3 domains— DBCI content (Figure 2B), DBCI delivery (Figure 2A), and context (Figures 2-5)—based on the conceptual framework for engagement with DBCIs by Michie and colleagues [6]. During coding, the researchers were blinded to the overall trial outcomes. All analyses were performed with Nvivo12 (QSR International).

### Triangulation and Synthesis of Quantitative and Qualitative Findings

All findings were integrated by matching the quantitative and qualitative results against the attributes and concepts—DBCI content, DBCI delivery, and context—of Michie’s conceptual framework (Figure 2) in a joint display for further comparisons and metainferences [6].

### Ethical Considerations

The SMARTDiabetes study was approved by the Peking University Health Sciences Institutional Review Board, Beijing, China, (IRB00001052-15062), and The University of Sydney Human Research Ethics Committee, Sydney, Australia (project no. 2016/105). The trial was registered at ClinicalTrials.gov (registration record NCT02726100, March 22, 2016). All participants provided written informed consent to the collection of individual-level trial assessment data and app usage data at the beginning of the trial. All interviewed participants provided verbal consent prior to interviews. All data were deidentified to ensure privacy and confidentiality. Participants were not financially compensated for taking part in the trial; however, all participants received free health examinations at baseline, midterm, and study completion.

## Results

### User Engagement

A total of 1038 participants from 40 clusters (20 rural villages and 20 urban communities) were assigned to the intervention arm. The mean MAUs proportion during the 16-month assessment period was 46.4% (SD 7.8%), with a gradual decline from 56.3% at the beginning (April 2018) to 42.2% at the end of follow-up (July 2019) (Figure 3). The proportion of sustained active users was 32.9% (342/1038 participants), with rural users showing a significantly higher rate than urban users (rural: 207/541 participants, 38.3% vs urban: 135/497 participants, 27.3%; *P*<.001).

After the initial period of training, installation, and familiarizing with the app, by May 2018, most users had been actively entering their FBG data into the app, with 98.9% (491/495) of rural and 87.4% (473/541) of urban users meeting the app-recommended monitoring frequencies (Figure 4). Throughout the entire trial period, the mean proportion of users meeting the recommended FBG-monitoring frequencies was 72.5% (SD 16.5%), with rural users outperforming their urban counterparts (rural 82.9%, SD 2.0% vs urban 61.6%, SD 3.6%; *P*<.001).

Table 1 summarized the monthly click-through rates per person for each of the 15 functions of the SMARTDiabetes app. The median total clicks per person per month was 3.9, with rural participants using more (rural 4.9 vs urban 3.5; *P*=.32). The most commonly used app functions were the 2 reminders “Diet punch card” and “Exercise punch card,” followed by the 2 self-monitoring ones “Blood glucose” and “Blood pressure,” all of which were incentivized functions.

**Table 1.** Number of clicks per person per month of each app function (1038 participants over 16 months of observation).

Functions	Urban (n=497)		Rural (n=541)		Total (N=1038)	
	Mean (SD)	Median (IQR)	Mean (SD)	Median (IQR)	Mean (SD)	Median (IQR)
Chronic disease self-monitoring						
Blood glucose (self-monitoring, goal setting, and feedback)^a^	1.9 (3.5)	0.6 (0.1- 2.3)	1.2 (1.9)	0.5 (0.1-1.7)	1.6 (2.8)	0.5 (0.1-1.9)
Blood pressure (self-monitoring, goal setting, and feedback)^a^	1.5 (3.0)	0.4 (0.1-1.6)	0.8 (1.5)	0.3 (0.1-0.9)	1.1 (2.3)	0.4 (0.1-1.2)
Body weight (self-monitoring and feedback)^a^	0.5 (1.0)	0.1 (0-0.5)	0.4 (1.0)	0.1 (0-0.6)	0.4 (1.0)	0.1 (0-0.6)
Blood lipid (self-monitoring, goal setting, and feedback)	0.2 (0.7)	0 (0-0.3)	0.3 (1.1)	0 (0-0.3)	0.3 (0.9)	0 (0-0.3)
Healthy lifestyle reminder						
Diet (reminder)^a^	3.0 (8.6)	0.7 (0-2.1)	3.1 (6.2)	0.3 (0-2.7)	3.0 (7.5)	0.5 (0-2.2)
Exercise (reminder)^a^	2.8 (5.8)	0.6 (0-2.0)	2.9 (5.7)	0.3 (0-2.7)	2.8 (5.8)	0.6 (0-2.3)
Health records						
Health status summaries	0.6 (1.8)	0.1 (0-0.4)	1.1 (3.6)	0.1 (0-0.8)	0.9 (2.9)	0.1 (0-0.6)
Healthy lifestyles (self-monitoring and feedback)	0.1 (0.3)	0 (0-0.1)	0.2 (0.5)	0 (0-0.1)	0.1 (0.4)	0 (0-0.1)
Medications (medical record)	0.1 (0.3)	0 (0-0.1)	0.1 (0.2)	0 (0-0.1)	0.1 (0.2)	0 (0-0.1)
Physical examinations and laboratory tests (medical record)	0.1 (0.3)	0 (0-0.1)	0.1 (0.2)	0 (0-0.1)	0.1 (0.3)	0 (0-0.1)
Diabetic complications (medical record)	0.1 (0.3)	0 (0-0.1)	0.1 (0.3)	0 (0-0.1)	0.1 (0.3)	0 (0-0.1)
Risk assessment						
iCVD^b^ risk assessments (feedback)^a^	0.4 (0.8)	0.1 (0-0.5)	0.5 (0.8)	0.1 (0-0.8)	0.5 (0.8)	0.1 (0-0.6)
Other						
Settings (system settings and reward system)^a^	1.2 (2.5)	0.3 (0-1.1)	1.0 (1.8)	0.3 (0.1-1.0)	1.1 (2.2)	0.3 (0-1.1)
Messages (communication)	0.7 (1.4)	0.1 (0-0.6)	0.9 (1.5)	0.2 (0-1.1)	0.8 (1.5)	0.1 (0-0.9)
Knowledge and skill bank (healthy literacy)	0.2 (0.5)	0 (0-0.2)	0.3 (0.5)	0.1 (0-0.3)	0.3 (0.5)	0 (0-0.3)
Total	13.4 (22.5)	3.5 (0.5-14.9)	12.9 (17.8)	4.9 (0.6-18.9)	13.2 (20.2)	3.9 (0.6-17.3)

a *Directly or indirectly incentivized functions.

b iCVD: ischemic cardiovascular diseases.

### Associations Between App Engagement and Effectiveness

As shown in Figure 5, sustained active users and users with FHP involvement achieved significantly better results in primary outcome and all 3 secondary outcomes. Users with the fewest clicks (click level 1) had a 31.0% success rate in achieving any 2 of the “ABC” goals. This rate remained unchanged at 31.3% in click level 2 (RR 1.03, 95% CI 0.86-1.25; *P*=.73). However, click level 3 users showed a statistically significant improvement, with a 36.4% success rate (RR 1.27, 95% CI 1.07-1.51; *P*=.006), and those in click level 4 achieved an even higher 44.9% success rate (RR 1.55, 95% CI 1.33-1.82; *P*<.001). This gradient in health outcomes across the different levels of user engagement, as measured by the total number of clicks on the SMARTDiabetes app functions, was also evident in achieving each individual “ABC” goal at 24 months.

### Rural-Urban Heterogeneity

Table 2 showed the disparities between rural and urban settings. Rural PHC facilities had fewer health care resources but were 100% (20/20) government-owned compared with 65% (13/20) of urban ones and were more distant from tertiary hospitals (13.3 km vs 1.9 km; *P*<.001). Rural participants were younger (*P*<.001) but had lower educational levels. Despite these differences, rural participants demonstrated higher engagement across all levels: a larger proportion of sustained active users (38.3% vs 27.2%; *P*<.001), greater FHP involvement (48.6% vs 21.5%; *P*<.001), more frequent interaction with the app (4.9 clicks per person per month vs 3.5; *P*=.32), and higher rates of self-monitoring FBG (82.9% vs 61.6%; *P*<.001).

**Table 2.** Rural-urban disparities.

Quantitative findings—characteristics	Urban	Rural	*P* value
PHC^a^ facilities characteristics			
Number of participating facilities in the intervention arm	20	20	N/A^b^
Government owned facilities, n (%)	13 (65)	20 (100)	.003
Size of the community served, median (IQR)	6366 (5024-7899)	2497.5 (2022-3651)	<.001
Distance to the nearest tertiary hospital (km), mean (SD)	1.9 (1.1)	13.3 (6.7)	<.001
Staff per facility, median (IQR)	6.0 (3.5-12.5)	2.0 (2.0-4.0)	<.001
Registered doctor	2.5 (1.5-5.0)	1.5 (1.0-2.0)	.005
Registered nurse	3.0 (1.5-4.0)	0 (0-0)	<.001
Staff providing NCD^c^ management services	3.0 (2.0-4.5)	1.5 (1.0-2.0)	<.001
Users baseline characteristics			
Number of participants in the intervention arm	497	541	N/A
Male, n (%)	217 (43.7)	232 (42.3)	.82
Age (years), mean (SD)	62.7 (6.7)	60.3 (7.4)	<.001
Education level, n (%)			
Primary school or lower	156 (31.4)	238 (44.0)	<.001
Junior high school	171 (34.4)	193 (35.7)	N/A
Senior high school	128 (25.8)	108 (20.0)	N/A
Junior college	28 (5.6)	1 (0.2)	N/A
Bachelor’s degree or higher	14 (2.8)	1 (0.2)	N/A
Engagement metrics			
Proportion of sustained active users, n/ N (%)	135/497 (27.2)	207/541 (38.3)	<.001
Proportion of users with FHP^d^ involvement, n/ N (%)	92/427 (21.5)	252/518 (48.6)	<.001
Cohabitation rate of FHPs and patients, n/N (%)	54/92 (58.7)	192/252 (76.2)	<.001
Total clicks per person per month, median (IQR)	3.5 (0.5-14.9)	4.9 (0.6-18.9)	.32
Mean proportion of FBG^e^ self-monitoring rate, mean (SD)	61.6% (3.6%)	82.9% (2.0%)	<.001

a PHC: primary health care.

b N/A: not applicable.

c NCD: noncommunicable disease.

d FHP: family health promoter.

e FBG: fasting blood glucose.

### Influencing Factors of User Engagement

The rapid expert review of the SMARTDiabetes app features (DBCI content) suggested that 6 main features, corresponding to previously proven effective BCTs [23], were very likely to have contributed to the success of the trial. These features included (1) the incentive system (Reward), (2) real-time feedback on individual result interpretation and suggestions for improvement (Feedback), (3) in-app reminder messages and healthy lifestyle punch card (Reminders), (4) setting goals and targets (Goal setting), (5) self-monitoring tools (Self-monitoring), and (6) support from FHPs (Social support features). The review of the SMARTDiabetes trial implementation activities (DBCI delivery) revealed several factors that have been proven effective [6], including partnerships with the local authorities (Credibility features), ongoing professional support from PHC providers (Professional support features), and a step-by-step, illustrative app instruction brochure (Guidance) (see Figure S3 in Multimedia Appendix 1 for the brochure). However, the complex installation process and frequent software updates were identified as major barriers to adoption and sustained engagement (Ease of use).

Twelve stakeholders (2 PHC providers, 7 patients, and 3 FHPs) were interviewed. The thematic analysis confirmed that rewards of nominal gifts, automated reminders, patient involvement in regular chronic disease self-monitoring, and adequate support from both FHPs and PHC providers were the most highly valued features of the SMARTDiabetes app. These features were perceived by the key stakeholders to be most influential in contributing to our app engagement. Furthermore, several key barriers to engagement were also identified in the interviews. First, a few patient users reported reluctance to involve their FHPs as it was perceived to lay an excessive burden to the FHPs, and this was more likely to occur if the FHPs had a busy daily life schedule or they lived far apart from the patient users. Technical difficulties related to the app installation and version updates were cited by several key stakeholders as a major barrier. Also, older participants, those with low literacy, and those inexperienced in smartphone use were the least likely to be active users. Qualitative findings also showed that in rural areas, the patient-doctor relationship is more personal and community-focused, PHC facilities are more committed to government initiatives, and FHPs are actively involved in patient care, whereas in urban areas, patients could bypass PHC facilities in favor of accessible tertiary hospitals.

### Synthesis of Findings

Based on the conceptual framework of DBCI engagement by Michie and colleagues [6,23], our team constructed this program logic to suggest potential influences and pathways contributing to user engagement and trial effectiveness, according to (1) DBCI content, (2) DBCI delivery, and (3) context. Table 3 shows the joint display of synthesized mixed methods findings where key quantitative findings were juxtaposed with illustrative interview quotes.

**Table 3.** Summary of the quantitative and qualitative findings.

Concept	Attribute	SMARTDiabetes features	Dose/fidelity	Quantitative evidence of effectiveness	Qualitative evidence of effectiveness
DBCI^a^ Content	Rewards	Users accumulate reward points by using certain functions in the app (Table S1 in Multimedia Appendix 1). Each user can earn a maximum of 100 points per month. These points can then be redeemed for nominal gifts (valued 5‐30 CNY^b^), distributed by their PHC^c^ providers. The reward system was launched in April 2018 and had been active throughout the rest of the trial.	In total, app users generated 982,005 reward points, with 93.2% (915,571/982,005) redeemed. In total, 97.0% (1006/1038) users have received gifts.	The incentivized app functions were more commonly accessed than those without (Table 1). After the introduction of the reward system in April 2018, there was a significant increase in the number of active users (Figure S4 in Multimedia Appendix 1). The rate of users meeting the recommended FBG^d^-monitoring frequencies increased from 77% to 99% in rural sites; and from 27% to 87% in urban sites in the following 2 months (Figure S3 in Multimedia Appendix 1).	"I am delighted to have earned the top-ranked reward points in my group” (Patient user, urban). “The monetary value of the reward isn’t important. The gift serves as motivation for making positive changes” (Patient user, urban). "The monetary value of the gift doesn’t matter. As long as they get something, it should be effective [to motivate patient engagement" (PHC provider, urban). "Patients will be happy even if they get the smallest gift, but they need to be able to redeem the gifts more frequently” (PHC provider, rural).
DBCI Content	Feedback	The app provided real-time feedback based on user inputs after users self-recorded their FBG readings.	The mean proportion of users meeting the recommended FBG self-monitoring and uploading frequencies was 72.5% (SD 16.5%).	N/A^e^	N/A
DBCI Content	Reminders	Users received in-app messages reminders to encourage self-monitoring of FBG and BP^f^, along with alerts for potential risks of unhealthy practices. The messages were categorized into 4 genres: urgent, serious, self-management, and system notifications. The “Diet” and “Exercise” functions set reminders to adhere to a healthy diet and daily exercise through a digital register.	In total, 57.7% (291/504) urgent messages, 14.3% (42,625/298,873) serious messages, 6.4% (35,103/550,912) self-management messages, and 15.1% (1565/10,341) system notifications were read by the users. A total of 58,581 click-throughs to the “Diet” function and 55,126 to the “Exercise” function (Table 1).	N/A	"The app sends reminders if you forget to measure your FBG or BP. When you receive the warning messages, it serves as a reminder, which is helpful, especially for elderly users who may forget at times. The “Diet” Da Ka (punch card) register advises me daily to consume less flour and starch, which I find beneficial. I’m particularly keen on the “Exercise” feature, where I can record my daily physical activity” (Patient user, urban).
DBCI Content	Goal setting	On the app’s main display screen, the latest FBG, BP, and LDL-c^g^ data were shown with a visually memorable color grading scheme: good results were marked in green, moderate ones in yellow, and poor ones in red (Figure 1A). The “ABC” goals were set to achieve FPG^h^ level below 7.0 mmol/L; BP readings below 140/80 mm Hg; and LDL-c levels below 100 mg/dL or 2.6 mmol/L.	Each time at login, users could review their 3 “ABC” goals displayed upfront on main screen.	N/A	N/A
	Self-monitoring	The app encourages users to self-monitor and upload their FBG and BP readings.	Throughout the trial, the mean frequency of FBG measurements was 1.50 (SD 0.39) times per month per person and BP levels were 1.45 (SD 0.39) times per month per person.	N/A	"I monitored my BP and FBG levels more frequently after using the app” (Patient user, urban). “I used the BP and FBG functions the most (to upload and record BP and FBG levels)” (Patient user, urban).
DBCI Content	Social support features	Family members played an active role in assisting patients with diabetes management.	In the intervention arm, 36.4% (344/945) of participant users reported having a family member who was able to assist them with the app (Table 2—Proportion of users with FHP^i^ involvement). The majority of FHPs were the patients’ adult children (78.8%), followed by their spouses (14.2%). Most FHPs were living together with the patient users (71.5%) (Table S3 in Multimedia Appendix 1).	FHP involvement was associated with better primary clinical outcomes (40.5% vs 33.4%; RR^j^ 1.30, 95% CI 1.17-1.44; *P*<.001), as well as better secondary clinical outcomes, including HbA_1c_^k^ (20.9% vs 16.6%; *P*=.02), BP (57.3% vs 54.4%; *P*=.04), and LDL-c (51.0% vs 43.9%, *P*<.001) (Figure 5). Of the various support and assistance provided by the patient users’ FHPs, reminding of medication uptake (66.0%), dietary control (64.8%), and exercise (57.3%) were the most common. This was closely followed by accompanying patient users to seek medical treatment when needed (50.3%) (Table S3 in Multimedia Appendix 1).	Facilitators: “FHPs supervise the patients. Sometimes they buy medicine for them” (PHC provider, rural). "My wife supported me in using this app. She learned how to use it first and then taught me. I am not good at using smartphones, and I tend to forget how to use the app” (Patient user, urban). “I reminded the patient to take the medication“ (FHP user, rural). Barriers: “My children are busy, so I don’t want to bother them” (Patient user, urban). “FHPs are busy” (PHC provider, rural). “My children come home about once per year” (Patient user, rural).
DBCI Delivery	Credibility features	The app and the brochure ( Figure S3 in Multimedia Appendix 1) displayed main local health authority’s (our study partners’) logos (the Hebei Medical University and Shijiazhuang Center for Disease Control and Prevention).	At each app login	N/A	N/A
	Professional support	An initial app induction about downloading, installation, and key functions was followed by continuous support from pretrained PHC providers.	PHC providers in the intervention arm were trained to download, install, update, and skillfully use every function of the app (“train the trainer”).	N/A	“The app made my communication with my doctor more frequent and more convenient. Our relationship became closer” (Patient user, urban). "I tend to forget how to use the app. I need help from the doctor so that I can continue to use the app” (Patient user, urban). "I gathered all patients and FHPs in my clinic and talked with them about the app. If they can’t make it, I went to organise other teaching chances at another time” (PHC provider, rural)*.*
DBCI Delivery	Guidance	A detailed, easy-to-understand instruction brochure (app operation manual), accompanied by a set of picture-based illustrative PowerPoint slides, was developed (Figure S1 in Multimedia Appendix 1).	Written app operation instruction materials were adequately distributed to all participating PHC providers, which further facilitated their patient user training.	N/A	N/A
	Ease of use	The app installation and updating process was perceived NOT to be easy to follow. Users were initially instructed to scan a QR code, which then directed them to go through a few steps to gain the system access permission before they could download.	App retraining had to be repeated every time there were major updates.	N/A	"Upgrading is challenging for patients as they need to download first and then find the file and obtain permission to access the app...It’s too complicated when the upgrades are very frequent” (PHC provider, urban). “I don’t understand the “iCVD risk” function as it is too complicated” (FHP user, rural).
**Context**	Population	Older people, those with low education literacy, and those with low smartphone experiences were the least likely to engage with the app.	N/A	The mean age of the patient users in the intervention arm was 61.4 years (SD 7.1), with 62.8% (652/1038) aged 60 years or older. Close to half (758/1038, 73.0%) of the patient users did not complete the Chinese 9-year compulsory education (ie, not completing senior high school).	“I feel that older people may struggle to use the app due to lower education levels, making it difficult for them to learn new skills. This is why patients in rural areas often rely more on their FHPs, while urban patients may not face the same challenges” (PHC provider, urban). “I am old, and it’s hard for me to accept new things. Our generation has only recently started using smartphones. We need to take it slowly” (Patient user, urban).
Context	Setting (rural vs urban)	While the SMARTDiabetes intervention demonstrated significant improvement in the proportion of participants achieving at least 2 American Diabetes Association “ABC” management targets compared with usual care (intervention group: 35.9% vs usual care: 29.9%; *P*=.025), we observed significant heterogeneity in the primary outcome between rural (intervention group: 42.6% vs usual care: 31.0%) and urban (intervention group: 27.9% vs usual care: 28.6%) settings (*P*=.02 for heterogeneity).	N/A	Refer to Table 2	Closer patient-doctor relationship in rural areas: In rural areas, the patient-doctor relationship often resembles that of close-knit neighborhoods, characterized by familiarity and reciprocity. In contrast, in urban areas, personal contact is often more distant, with the patient-doctor relationship largely shaped by the PHC provider’s professional expertise and communication skills. Stronger commitment of PHC facilities in rural areas: Government-owned or public-funded PHC facilities tend to show greater commitment to government-endorsed initiatives, while private-owned facilities, more common in urban areas, often prioritize profit-driven activities. Bypassing the PHC facilities in urban areas: In urban areas, where tertiary hospitals are much closer, patients often bypass PHC facilities, opting instead to seek health care directly at nearby hospitals without needing a referral. This practice potentially undermines the effectiveness of PHC providers in delivering continuous care to patients with chronic conditions. More FHP involvement in the rural areas: FHPs, primarily patients’ children and spouses, played a vital role in patient care. They assisted with app use, including reminding patients to take medications, supporting dietary management, encouraging regular exercise, and often accompanying patients’ hospital visits for scheduled medical appointments. While both rural and urban patients reported reluctance to ask for help from FHPs, rural patients generally found it easier due to their closer proximity to family members.

a DBCI: digital behavior change intervention.

b 1 CNY = US $0.1514, based on the average conversion rate in 2018.

c PHC: primary health care.

d FBG: fasting blood glucose.

e N/A: not applicable.

f BP: blood pressure.

g LDL-c: low-density lipoprotein cholesterol.

h FPG: fasting plasma glucose.

i FHP: family health promoter.

j RR: relative risk.

k HbA_1c_: glycated hemoglobin.

## Discussion

### Principal Findings

The SMARTDiabetes trial was the first long-term (over 1 year) mobile app intervention for T2DM self-management conducted in a real-world PHC setting. Throughout the whole trial period, 46.4% of participants were MAUs and 32.9% (342/1038) were sustained active users. We observed a dose-response relationship between user engagement and trial effectiveness. Specifically, sustained engagement with the app, involvement of family members, and frequent interaction with app functions were associated with improved clinical outcomes. Key drivers of user engagement and trial effectiveness included 6 known BCTs underpinning the 15 app functions (ie, reward, feedback, reminders, goal setting, self-monitoring, and social support features), 4 implementation facilitators (credibility features, professional support, guidance, and ease of use), and 5 contextual contributors (younger age, better education, greater smartphone literacy, closer patient-doctor relationship, and stronger PHC facilities commitment).

We observed a modest yet effective level of engagement with the SMARTDiabetes app, with half of the participants logging in at least once per month, leading to significant improvement in achieving at least 2 ADA “ABC” management targets [5]. Effective engagement with DBCI is empirically defined as the level of interaction necessary to achieve the intended health outcomes [11]. It involves both “microengagement,” which refers to the intensity and consistency of app use, and “macroengagement,” which pertains to the alignment with and commitment to the broader intervention goals. Both forms of engagement are closely intertwined, and while each is important, neither alone is sufficient for achieving effective outcomes. Previous DBCIs for diabetes primarily focused on patients and doctors only [12-15]. However, the SMARTDiabetes mHealth system, consisting of 2 apps and 1 website, fostered stronger connections among patients, family members, PHC providers, and local governors ( Figure S1 in Multimedia Appendix 1). It enabled remote family members to stay informed about their loved ones’ health and facilitated lay FHPs in delivering evidence-based, guideline-informed care (eg, FHPs helped remind patients to take medications, supported dietary management, encouraged regular exercise, and accompanied patients to medical appointments; Table S3 in Multimedia Appendix 1). The system also encouraged patients to actively engage with their doctors through in-app messages, phone calls, and face-to-face interactions during training and the gift redemption process, addressing a key barrier to diabetes management at the PHC level in China [21]. Additionally, it provided governors with real-time access to validated health statistics and powerful data visualization tools, enabling them to track changes, make informed comparisons, and drive performance-based improvements in the quality of care. These macrolevel improvements in engagement were hard to measure yet indispensable to the trial’s overall success.

Comparing engagement levels across DBCI studies is challenging and often inconclusive due to the heterogeneity in intervention types, implementation duration, delivery modes, population demographics, study settings, intended behavioral objectives, and measures of engagement [24]. Standardized checklists to describe engagement with DBCIs will assist with comparing engagement across studies. The observed nonengagement rate (170/1038, 16.4%) in our study compares favorably with previous studies, which reported nonengagement rates of 50.2% (101/201) [14] and 57.2% (16,958/29,643) [25]. The SMARTDiabetes trial’s user engagement compared favorably at 6, 9, and 12 months to 3 similar studies conducted in real-world settings with large populations—the Cornerstones4Care study reported a 30.5% engagement rate in 6 months [25], the National Health Service Digital Diabetes Prevention Programme study had a 36.5% engagement rate in 9 months [26], and the myDESMOND study showed a 17.6% engagement rate in 12 months [27].

The decline in app engagement over time has been commonly reported in mHealth interventions [18,28]. We also noticed a gradual decline in engagement in our study, with the proportion of MAUs decreasing by 25.0%, from 56.3% to 42.2% over 16 months, resulting in a retention rate of 75.0%. In comparison, the Cornerstones4Care study reported a 71.4% (9051/12,685) retention rate over 6 months [25], the National Health Service Digital Diabetes Prevention Programme study showed a 54.2% (667/1230) retention rate over 9 months [26], and the myDESMOND study had a 31.3% (1676/5360) retention rate over 12 months [27].

Previous research has demonstrated a strong link between user engagement with DBCIs and their effectiveness in achieving intended health outcomes [9,29]. The dose-response relationship between the total number of app clicks and improvements in the primary outcome suggests that DBCIs require a minimum threshold of engagement—an optimal dose—to achieve intended outcomes [6]. Beyond this threshold, the intensity of engagement has a positive correlation with the extent of outcome improvements. However, due to the complexity of engagement, defining the optimal dose is challenging, as some individuals may require a longer period of interaction with the app than others [30]. It is also plausible that the individuals who are more motivated and successful in achieving behavioral change tend to engage more, or that those who engage more differ systematically from those who are less engaged [29]. Assessing the extent to which engagement influences behavioral and health-related outcomes is challenging, as it may be confounded by factors such as increased motivation and self-regulation skills [11]. Future research should investigate these potential confounders to draw more definitive conclusions.

The design of app features (ie, DBCI content) plays a crucial role in user engagement, and 6 effective BCTs were repeatedly associated with higher engagement levels [6,9]. In our study, we found that incentive features (ie, “rewards”) [11] were highly effective both in boosting engagement at the initial adoption phase (ie, “trigger”) and in sustaining long-term use. Our findings also suggest that the impact of rewards is believed to rely less on their monetary value and more on their distribution frequencies (instant positive reinforcement). The most frequently used functions were the diet and exercise punch cards, both of which integrated key aspects of gamification and loyalty. Gamification is a promising strategy for enhancing user engagement [31], and the commercial sector has excelled in implementing these concepts. For instance, Duolingo has used game elements such as leaderboard, level system, and badges to motivate users, achieving a staggering 100 million MAUs [32]. Academia can benefit from studying these successful models to improve the design of DBCIs.

One important lesson we learned was that the SMARTDiabetes app was not user-friendly when it came to downloading, installing, and updating. The app was unavailable on public app markets, requiring users to scan a QR code and download it via a third-party website. After downloading, users had to manually grant permissions for the app and complete the installation process. Moreover, with every update, users were forced to repeat the entire procedure. This cumbersome process likely contributed to a significant loss of active users [17]. Future mobile app interventions should focus on integrating the installation and updating processes with commonly used app stores.

Finally, context matters in implementation science research [33]. Our previous study showed that the same mHealth intervention package—using identical intervention tools and delivery strategies—was significantly more effective in rural settings than in urban settings [5]. The quantitative findings of this process evaluation showed that rural participants demonstrated higher engagement with the SMARTDiabetes program, reflected in a higher proportion of sustained active users (38.3% vs 27.2%; *P*<.001), more frequent app interactions (4.9 clicks per person per month vs 3.5; *P*=.32), and a higher rate of FBG self-monitoring (82.9% vs 61.6%; *P*<.001). Our qualitative findings indicated that higher engagement in rural settings was attributed to 3 main factors: closer and more reciprocal patient-doctor relationships, stronger commitment from PHC facilities, and greater involvement from the family members (Table 3). Together, these factors created a more supportive environment for patients’ sustained participation in the SMARTDiabetes program.

### Strengths and Limitations

The strength of this study is its mixed methods design to capture multifaceted engagement with DBCIs. Study limitations include the following: First, the study’s geographical scope was limited to Northern China, potentially constraining the generalizability of its findings to this specific health system context. However, this region’s PHC infrastructure and services, based on the National Essential Public Health Service Programme, are representative of many areas across China. Consequently, we posit that these findings offer valuable insights applicable to other regions. Second, the total number of interviews conducted was less than anticipated, due to the difficulties of face-to-face interaction during the COVID outbreaks. Consequently, the views expressed by limited participants may not be representative of the total study population. Third, since the study was conducted a few years ago, there may have been changes over time. However, the expected impact of such changes is likely minimal, given that the core policies and regulations for diabetes and hypertension management have remained unchanged since 2009.

### Conclusions

Long-term, sustained engagement with a mobile app intervention for T2DM self-management is achievable in the PHC setting in China. Our study reveals a dose-response relationship between user engagement with DBCIs and their effectiveness in achieving intended health outcomes. Specifically, DBCIs require a minimum level of engagement to be effective. The factors influencing engagement identified in the SMARTDiabetes study provide valuable insights for developing targeted strategies to enhance user engagement and improve overall program effectiveness.

## Supplementary material

10.2196/76699 Multimedia Appendix 1 Intervention design, app algorithms, participant characteristics, and study implementation.

10.2196/76699 Checklist 1 GRAMMS (Good Reporting of a Mixed Methods Study) checklist.

## Abbreviations

BCT: behavior change technique
BP: blood pressure
DBCI: digital behavior change intervention
FBG: fasting blood glucose
FHP: family health promoter
HbA_1c_: glycated hemoglobin
iCVD: ischemic cardiovascular disease
MAU: monthly active user
mHealth: mobile health
PHC: primary health care
RR: relative risk
T2DM: type 2 diabetes mellitus
