# Supplementary material for: Real-World Long-Term Engagement With a Mobile App Intervention to Improve Self-Management of Type 2 Diabetes Mellitus in China (SMARTDiabetes): Mixed Methods Study
Source: JMIR Mhealth Uhealth. 2026 Mar 3;14:e76699. doi: 10.2196/76699 (PMC12978905; doi:10.2196/76699)
Supplement: Multimedia Appendix 1 [file mhealth-v14-e76699-s001.docx]

**Multimedia Appendix 1**


Table S1. User engagement incentives.

| **Functions** | **Score** | **Maximum score in each month** |
| --- | --- | --- |
| Healthy Diet Punch Card | 2 points per session | 10 |
| Exercise Punch Card | 2 points per session | 10 |
| Risk Assessment Quiz | 10 points per session | 10 |
| Fasting Blood Glucose (FBG) Measurement | 10 points per entry | 10 |
| Blood Pressure (BP) Measurement | 10 points per entry | 10 |
| Weight Measurement | 10 points per entry | 10 |
| FBG Reading Result ^a^ | Poor-5 points | 20 |
|  | Moderate-10 points |  |
|  | Good-20 points |  |
| BP Reading Result ^b^ | Poor-5 points | 20 |
|  | Moderate-10 points |  |
|  | Good-20 points |  |
| **Total** | **-** | **100** |

*a: Refer to Table A3 as good, moderate, and poor FBG results.*

*b: Refer to Table A3 as good, moderate, and poor BP results.*

Table S2. FBG and BP result interpretation and monitoring frequency algorithm.

| **App algorithm** | **Result** | **Monitoring frequency** |
| --- | --- | --- |
| FBG |  |  |
| IF 4.4≤FBG≤7.0 | Good | Check again in 4 weeks |
| IF 7.1≤FBG≤9.8 | Moderate | Check again in 2 weeks |
| IF FBG≤4.3 or FBG≥9.9 | Bad | Check again in 1 week |
| BP |  |  |
| IF 90≤SBP<120 AND 60≤DBP<80 | Good | Check again in 4 weeks |
| IF SBP<90 OR DPB<60 | Moderate | Check again in 2 weeks |
| IF 120≤SBP<160 OR 80≤DBP<100 | Moderate | Check again in 2 weeks |
| IF SBP≥160 OR DPB≥100 | Bad | Check again in 1 week |

*Note: FBG = fasting blood glucose; SBP = systolic blood pressure; DBP = diastolic blood pressure*

Table S3. Main FHPs’ characteristics.

| **N (%)** | **Urban (N = 92)** | **Rural (N = 252)** |
| --- | --- | --- |
| Age range |  |  |
| <30 years | 7 (7.6%) | 26 (10.3%) |
| 30-59 years | 72 (78.3%) | 198 (78.6%) |
| ≥60 years | 13 (14.1%) | 28 (11.1%) |
| Gender |  |  |
| Male | 62 (67.4%) | 144 (57.1%) |
| Education completion |  |  |
| Junior high school or below | 24 (26.43%) | 103 (40.1%) |
| Senior high school | 31 (34.1%) | 84 (33.3%) |
| Junior college | 11 (12.1%) | 22 (8.7%) |
| Bachelor's degree or higher | 25 (27.5%) | 45 (17.9%) |
| Relationship with patients |  |  |
| Children | 74 (80.4%) | 197 (78.2%) |
| Spouses | 17 (18.5%) | 32 (12.7%) |
| Other | 1 (1.1%) | 23 (9.1%) |
| Assistance type |  |  |
| Reminding medication uptake | 46 (59.0%) | 181 (82.3%) |
| Supporting diet management | 51 (65.4%) | 172 (78.2%) |
| Encouraging exercise | 46 (59.0%) | 151 (68.6%) |
| Accompanying hospital visits | 39 (50.0%) | 134 (60.9%) |
| Promoting health literary | 23 (29.5%) | 89 (40.5%) |
| Assisting with blood glucose test | 20 (25.6%) | 67 (30.5%) |
| Assisting with insulin injection | 8 (10.3%) | 9 (4.1%) |
| Living arrangement |  |  |
| Live together | 54 (58.7%) | 192 (76.2%) |
| Mobile phone operating system |  |  |
| Android | 76 (82.6%) | 238 (94.4%) |
| IOS | 16 (17.4%) | 18 (7.1%) |


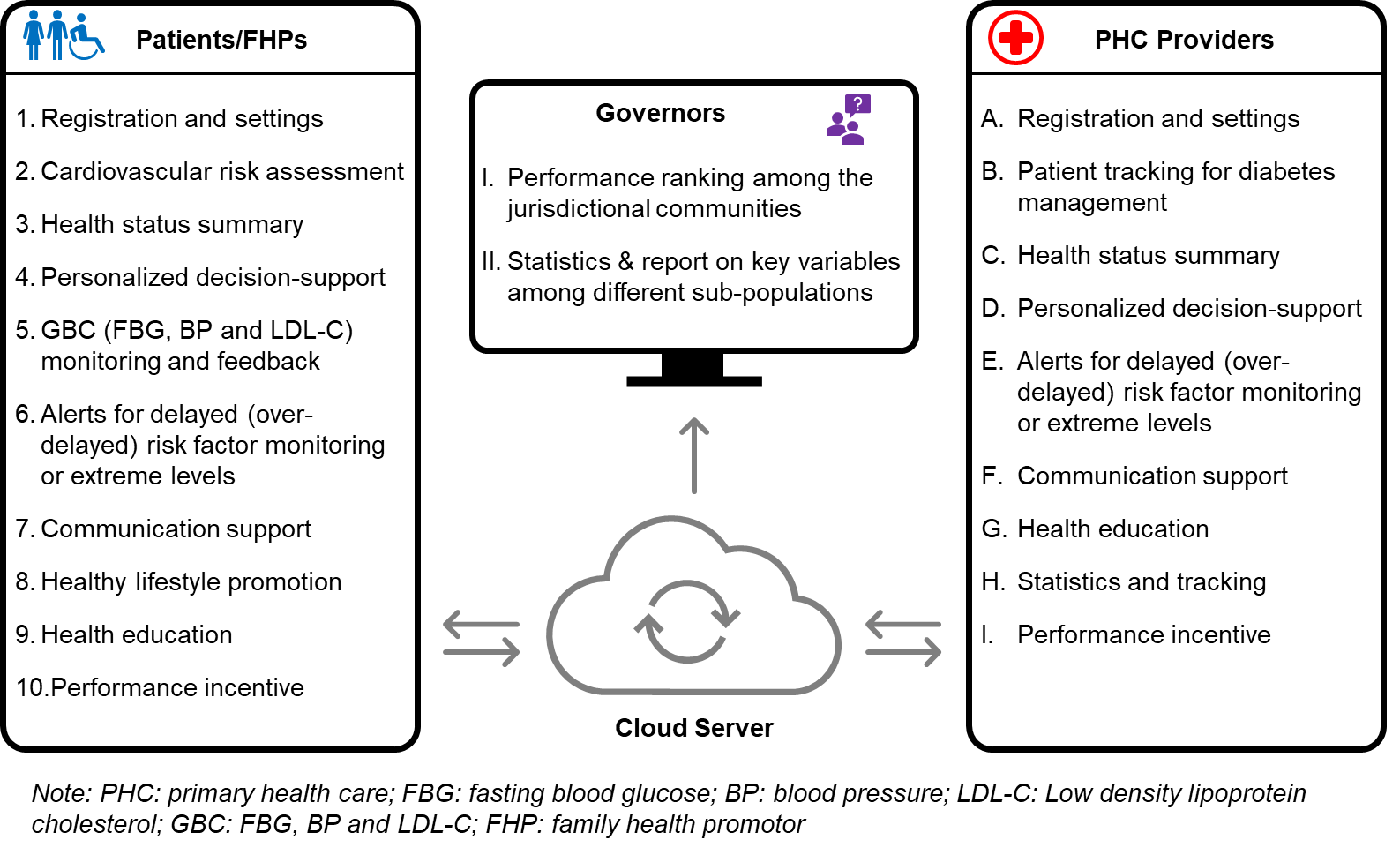


Figure S1. DBCIs of the SMARTDiabetes study.


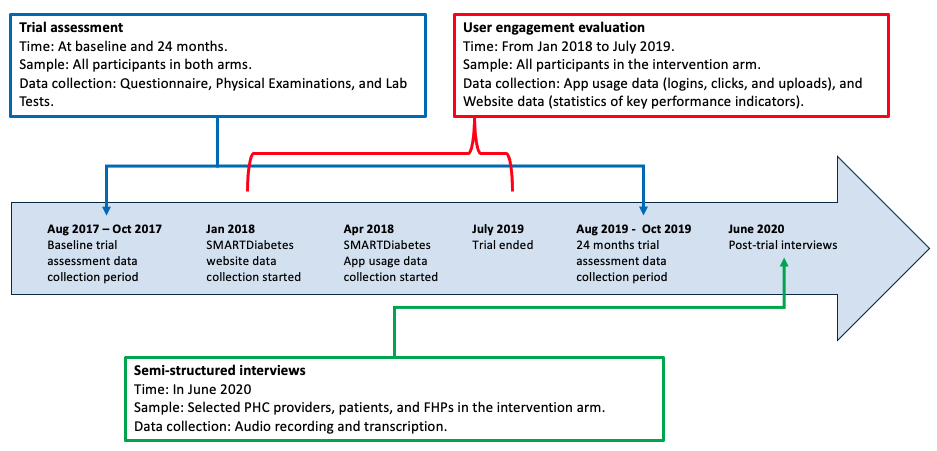


Figure S2. Timeline of the SMARTDiabetes Study data collection.


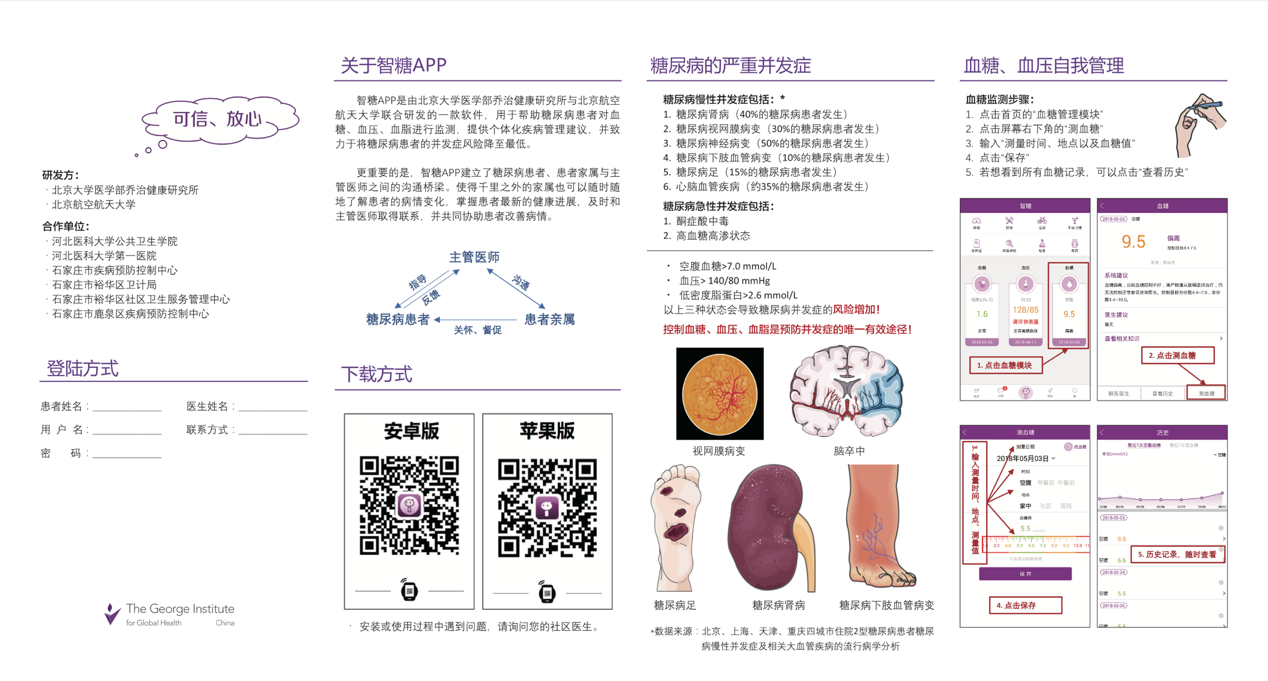


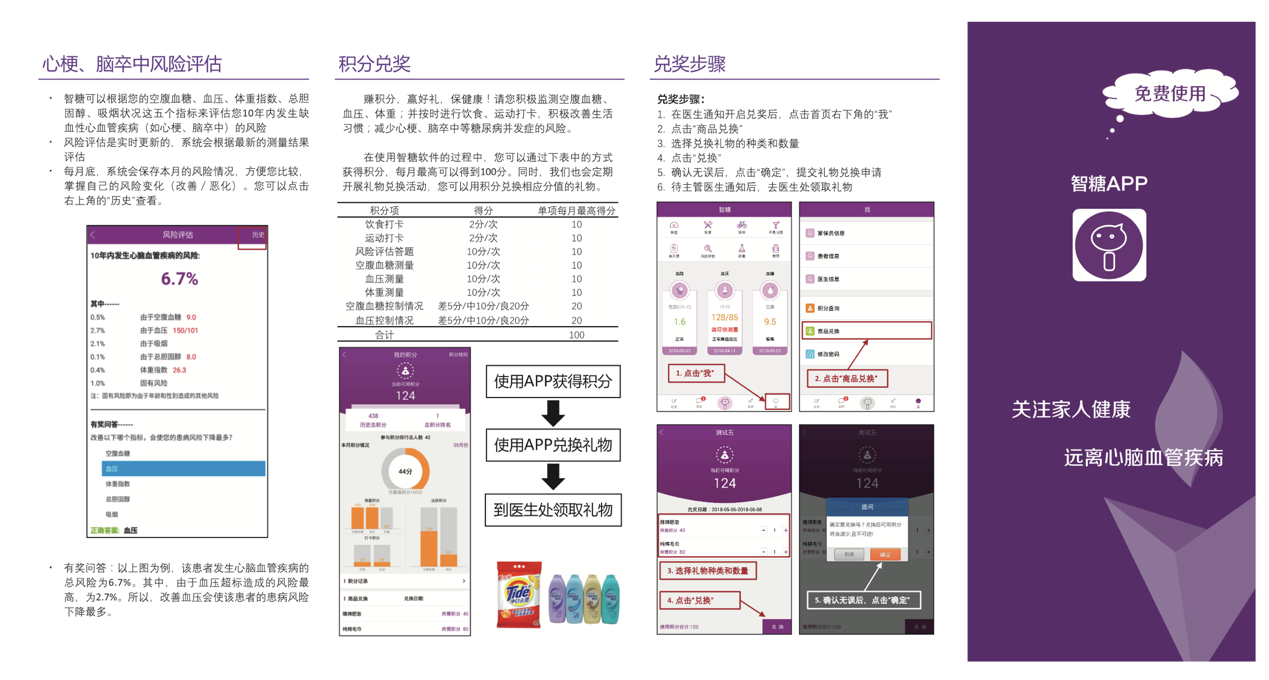


Figure S3. SMARTDiabetes app instruction brochure.

Figure S4. SMARTDiabetes adoption phase (Jan 2018 to May 2028).

**Posttrial Interview Guide (for PHC providers)**

**Before interview:**

1. Ice-breaking.
2. Introducing the aims of this interview.
3. Reminding of the audio recording and the protection of privacy.

**Interview Question:**

1. PHC Provider Demographics – [Age, Gender, Education, Computer literacy]

How would you rate your proficiency in using digital devices like computers and smartphones?

1. PHC Provider Psychological – [Beliefs, Motivation, Expectation, Self-efficacy]

What are the strengths and weaknesses of the SMARTDiabetes app?

If a mature mHealth management product were available in the future, would you opt to use or avoid this management model?

1. PHC Provider Setting – [Access, Resources, Time, Location, Policy, The System]

Have you encountered any problems with network connectivity or the SMARTDiabetes software? How have software updates affected your user experience, if at all?

How do you rate the equipment and test strips provided by the project? If they are unavailable, can the project still operate normally?

How much time do you typically devote to using SMARTDiabetes app? Does using the app interfere with your other work responsibilities?

Does your work location affect your ability to manage patients using SMARTDiabetes?

Does the health policy at your workplace and in your local area have any impact on managing patients using SMARTDiabetes?

Overall, in your opinion, how have your supervisors, work organization, and management structure facilitated or hindered your participation in the SMARTDiabetes project?

1. Service Users Context – [Demographic, Psychological, Setting]

In your opinion, what are the primary factors influencing patients and FHPs adherence to treatment? How can we effectively motivate patients and FHPs to get involved?

1. DBCI Delivery – [Barrier, Facilitator]

What are your thoughts on the PHC providers’ meetings and gift redeeming activities?

How did you reach out to/ communicate with patients and FHPs? How did you teach them to use the app?

1. DBCI Content – [Feedback, Reward, Ranking]

Could you briefly describe your typical approach to managing patients using the SMARTDiabetes app?

Which features of the SMARTDiabetes platform do you find most valuable or appealing?

1. Mechanism of Action – [Attitudes, Beliefs, Knowledge, Motivation, Skills]

What do you believe is the most crucial factor in facilitating behavior change among patients?

In what ways does the SMARTDiabetes platform enable patients to improve their health outcomes?

**Post-trial Interview Guide (for patients and FHPs)**

**Before interview:**

1. Ice-breaking.
2. Introducing the aims of this interview.
3. Reminding of the audio recording and the protection of privacy.

**Interview Question:**

1. User Demographics – [Age, Gender, Education, Computer literacy]

How would you rate your proficiency in using digital devices like computers and smartphones?

1. User Psychological – [Beliefs, Motivation, Expectation, Self-efficacy]

What are the strengths and weaknesses of the SMARTDiabetes app?

If a mature mHealth management product were available in the future, would you opt to use or avoid this management model?

What motivated you to use the SMARTDiabetes app (incentives, ranking, health goal, social support)?

1. User Setting – [Access, Resources, Time, Culture, Norm]

Have you encountered any problems with network connectivity or the SMARTDiabetes software? How have software updates affected your user experience, if at all?

How much time do you usually spend on the SMARTDiabetes app?

How was the involvement of FHP?

1. DBCI Content – [Feedback, Goal setting, Reminders, Self-monitoring, Reward, Ranking]

Which features of SMARTDiabetes have you used?

Which feature do you like the most? Why?

Which features have you not used? Why?

Any suggestions of improvement?

1. DBCI Delivery – [Complexity, Ease of use, Guidance, Professional support, Interactivity]

How was the teaching process?

Were there any difficulties in using it?

1. Mechanism of Action – [Attitudes, Beliefs, Knowledge, Motivation, Skills]

What changes have occurred in your disease management? How about SMARTDiabetes? What role did it play?

Does the SMART Diabetes project change your relationship and the way you communicate with your doctor? How?

Do you have a designated FHP? If yes, continue. If no, why not? What difficulties have you encountered?

Please describe the way your FHP uses the SMARTDiabetes app and how does your FHP help with your disease management?
