# Supplementary material for: Real-World Long-Term Engagement With a Mobile App Intervention to Improve Self-Management of Type 2 Diabetes Mellitus in China (SMARTDiabetes): Mixed Methods Study
Source: JMIR Mhealth Uhealth. 2026 Mar 3;14:e76699. doi: 10.2196/76699 (PMC12978905; doi:10.2196/76699)
Supplement: Checklist 1 [file mhealth-v14-e76699-s002.docx]

Checklist 1. Good Reporting of a Mixed Methods Study (GRAMMS) checklist.

| **Guideline** | **Page** |
| --- | --- |
| 1. Describe the justification for using a mixed methods approach to the research question. | 7 and 8 |
| 2. Describe the design in terms of the purpose, priority, and sequence of methods. | 7 and 8 |
| 3. Describe each method in terms of sampling, data collection and analysis. | 7 to 17 |
| 4. Describe where integration has occurred, how it has occurred and who has participated in it. | 15 and 16 |
| 5. Describe any limitation of one method associated with the present of the other method. | 35 |
| 6. Describe any insights gained from mixing or integrating methods | Table 3 and Discussion |
